# Association between first-line antidepressant use and risk of dementia in older adults: a retrospective cohort study

**DOI:** 10.21203/rs.3.rs-3266805/v1

**Published:** 2023-09-13

**Authors:** Hsin-Min (Grace) Wang, Wei-Han Chen, Shao-Hsuan Chang, Tianxiao Zhang, Hui Shao, Jingchuan Guo, Wei-Hsuan Lo-Ciganic

**Affiliations:** University of Florida; University of Florida; University of Florida; University of Florida; Emory University; University of Florida; University of Florida

**Keywords:** Antidepressants, psychotherapy, depression, dementia, older adults

## Abstract

**Background:**

Prior studies suggested that antidepressant use is associated with an increased risk of dementia compared to no use, which is subject to confounding by indication. We aimed to compare the dementia risk among older adults with depression receiving first-line antidepressants (i.e., SSRI/SNRI) versus psychotherapy, which is also considered the first-line therapy for depression.

**Methods:**

This retrospective cohort study was conducted using the US Medical Expenditure Panel Survey from 2010 to 2019. We included adults aged ≥50 years diagnosed with depression who initiated SSRI/SNRI or psychotherapy. We excluded patients with a dementia diagnosis before the first record of SSRI/SNRI use or psychotherapy. The exposure was the patient’s receipt of SSRI/SNRI (identified from self-report questionnaires) or psychotherapy (identified from the Outpatient Visits or Office-Based Medical Provider Visits files). The outcome was a new diagnosis of dementia within 2 years (i.e., survey panel period) identified using ICD-9/ICD-10 codes from the Medical Conditions file. Using a multivariable logistic regression model, we reported adjusted odds ratios (aORs) with 95% confidence intervals (CIs). We also conducted subgroup analyses by patient sex, age group, race, severity of depression, combined use of other non-SSRI/SNRI antidepressants, and presence of underlying cognitive impairment.

**Results:**

Among 2,710 eligible patients (mean age= 61±8, female=69%, white=84%), 89% used SSRIs/SNRIs, and 11% received psychotherapy. The SSRI/SNRI users had a higher crude incidence of dementia than the psychotherapy group (16.1% vs. 12.7%), with an aOR of 1.39 (95% CI=1.21–1.59). Subgroup analyses yielded similar findings as the main analyses, except no significant association for patients who were black (0.75, 95% CI=0.55–1.02), had a higher PHQ-2 (1.08, 95% CI=0.82–1.41), had concomitant non-SSRI/SNRI antidepressants (0.75, 95% CI=0.34–1.66), and had underlying cognitive impairment (0.84, 95% CI=0.66–1.05).

**Conclusions:**

Our findings suggested that older adults with depression receiving SSRIs/SNRIs were associated with an increased dementia risk compared to those receiving psychotherapy.

## BACKGROUND

One out of ten older adults aged ≥ 65 years suffers from dementia in the United States (US), and the prevalence dramatically increases with age.(1) The economic burden of dementia is estimated to be high, exceeding $321 billion (not including $272 billion in unpaid caregiving).(1) As such, dementia is among the leading contributors to the global disease burden, which accounts for 4.3% of the number of years lost due to ill health, disability, or early death (i.e., disability-adjusted life years). Furthermore, depression affects approximately 8.4% of US adults,(2) especially those aged 15–49 years.(3) Patients with early-life depression (i.e., onset before the age of 60) have a 2- to 3-fold higher risk of developing dementia,(4) probably through cerebrovascular changes, an increase in glucocorticoids and proinflammatory cytokines, and a decrease in nerve growth factors that lead to hippocampal atrophy and cognitive impairment.(5, 6)

Psychotherapy and antidepressants are considered the mainstay treatments for depression.(7) Selective serotonin reuptake inhibitors (SSRI, e.g., fluoxetine) and serotonin norepinephrine reuptake inhibitors (SNRI, e.g., venlafaxine) are considered first-line pharmacotherapy due to fewer side effects compared to other antidepressants.(8) Existing evidence regarding the association between antidepressants and dementia has been inconsistent. For example, Eisch et al. suggested that antidepressants may also have cognitive benefits owing to their anti-inflammatory and neurogenic properties in addition to reducing depressive symptoms.(9) Jacob et al. also found that the use of antidepressants was associated with a reduced risk of dementia in patients with moderate or severe depression compared to nonusers of antidepressants.(10) In contrast, Kodesh et al. suggested that antidepressant use was associated with a more than 3-fold increased risk of dementia compared to nonusers among older adults, probably due to their anticholinergic side effects.(11)

Prior studies are limited by only comparing antidepressant exposure with no exposure, which may be subject to confounding by indication and severity.(12) That is, patients taking antidepressants are likely to suffer from more severe depression than nonusers, while depression itself can be a risk factor for dementia, making separating the drug effect from depression severity challenging.(12) In addition, tricyclic antidepressants (TCAs, e.g., amitriptyline) are currently second-line pharmacotherapy for depression due to their higher anticholinergic burden that may increase multiple side effects (e.g., cognitive decline).(13) It may not be appropriate to combine all the classes of antidepressants into one group when evaluating the risk of dementia. Therefore, we aimed to compare the risk of dementia among older adults using SSRIs/SNRIs only versus those on psychotherapy only, adjusting for patients’ demographics, socioeconomic status, comorbidities, comedications, and most importantly, the severity of depression to minimize confounding by indication.

## METHODS

### Data source

This study used the 2010–2019 US Medical Expenditure Panel Survey (MEPS) data, a longitudinal, large-scale survey of noninstitutionalized adults in the US.(14) Each panel covers a two-year period, in which each surveyed household was interviewed five rounds. This survey encompasses information such as individual sociodemographic characteristics, disease diagnoses, comorbidities, and medication use. We selected and merged data from the full-year consolidated file, prescribed medicines file, medical conditions file, and outpatient visits file.

MEPS data is reviewed and approved by the Westat Institutional Review Board (IRB) annually and is established under a multi-project assurance (MPA M-1531) granted by the Office for Protection from Research Risks. After carefully removing individual’s identifiable information, an annual series of Public Use Files of de-identified MEPS data are made publicly available to researchers (https://meps.ahrq.gov/mepsweb/). Due to the nature of de-identification and public availability of the MEPS data, the University of Florida IRB determined the study exempt and did not require informed consent to participate.

### Study design

**Figure 1:**
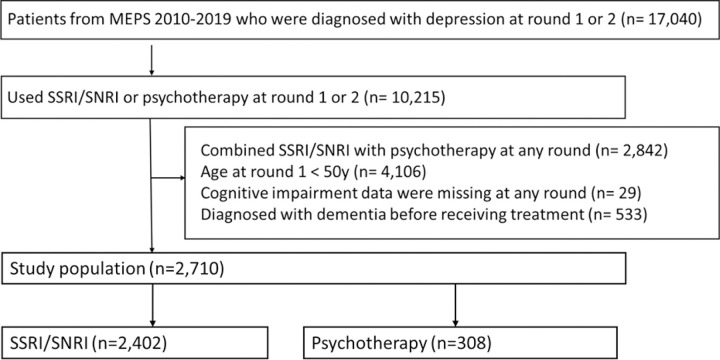
Study population selection. Abbreviation: MPES, Medical Expenditure Panel Survey; SSRI: selective serotonin reuptake inhibitors, SNRI: serotonin and norepinephrine reuptake inhibitors From the 2010–2019 MEPS data, we identified 17,040 patients diagnosed with depression at round 1 or 2, of which 10,215 patients used SSRI/SNRI at round 1 or 2. Each panel in MEPS data includes 5 rounds within 2 years. The reason for limiting to the first two rounds was to allow at least one year follow-up time for each patient. We excluded patients combining SSRI/SNRI with psychotherapy at any round (n=2.842), aged <50 years at round 1 (n=4106), missed cognitive impairment data at any round (n=29), and diagnosed with dementia before the index round (n=533). There were 2,710 patients included in out analytical cohort, with 2,402 (89%) using SSRIs/SNRIs and 308 (11%) receiving psychotherapy.

We conducted a retrospective cohort study restricted to adults aged ≥ 50 years with a depression diagnosis in round 1 or 2 of a two-year panel period to allow at least a 1-year follow-up time. Depression was identified using International Classification of Disease (ICD) codes (ICD-9: 296.20–296.25, 296.30–296.35, 300.4, 311; positive predictive value [PPV] = 92.0%; ICD-10: F32.0–32.9, F33.0–33.3, F33.8, F33.9, F34.1 & F41.2; PPV = 91.1%).(15) We further included those receiving SSRIs/SNRIs or psychotherapy at rounds 1 or 2, with an index round defined as the round when the first SSRI/SNRI or psychotherapy was prescribed. We excluded patients who (1) concomitantly used SSRIs/SNRIs and psychotherapy at any round, (2) had missing cognitive impairment data at all rounds during 2010–2019, and (3) had a dementia diagnosis before the index round. We followed up patients until the dementia outcome occurred or the end of each survey panel period (i.e., two years). Figure 1 depicts the details of the study cohort selection.

### Exposure and covariate ascertainment

Our exposure of interest was the patient’s receipt of SSRIs/SNRIs versus psychotherapy. We identified SSRI/SNRI use from the questionnaires in the prescribed medicines files using therapeutic classification variables from Cerner Multum, Inc. (Multum Lexicon Variables: TCnSn and TCnSn_n). Psychotherapy used was identified from the Outpatient Visits or Office-Based Medical Provider Visits files. Using the questionnaires in Full-Year Consolidated files, we measured covariates including demographic information (age, sex, race, insurance type, marital status, region, poverty, education, Patient Health Questionnaire (PHQ)-2 score, and cognitive impairment), lifestyle factors (smoking and physical activity), and access to healthcare information (i.e., delayed or unable to obtain necessary medical care/prescribed medications). The PHQ-2 score was used to estimate the severity of depression, which assessed the frequency of depressed mood and anhedonia over the past two weeks.(16) We also included covariates of comorbidities (cancer, type 2 diabetes, hyperlipidemia, hypertension, ischemic stroke, chronic heart disease, osteoarthritis, Parkinson’s disease, anxiety, sleep disorder, schizophrenia, and bipolar disorder) and other medication use (analgesics, benzodiazepines, anxiolytics, sedatives, hypnotics, non-SSRI/SNRI antidepressants, antipsychotics, and antiparkinsonian agents) that were extracted from the medical conditions files and prescribed medicines files, respectively (**Appendix** Table 1).

### Outcome ascertainment

We identified the outcome of interest, a new diagnosis of dementia within each two-year survey panel period, using ICD-9 codes (290, 331.0, 331.1, 331.2, 331.82, 331.83, 331.9, 438.0, 780.93) and ICD-10 codes (F00, F01, F03, F04, G30, G31.0, G31.1, G31.8, G31.9, I69.91, R41) from the Medical Conditions file (**Appendix** Table 1). The PPV values of using these ICD codes to identify dementia ranged from 73.2 to 93.6%.(15)

### Statistical analysis

Given that the MEPS uses a complex survey design with clustering, stratification, and weights, we conducted all the analyses using the survey procedure in SAS version 9.4 (SAS Institute, Inc., Cary, NC).

The statistical analysis for this study comprised the following steps. First, we excluded the covariate “type of insurance” since its missingness was too high (90.8%). The proportion of missing information varied from 0.1–28.2% across the remaining variables. We used a multiple imputation approach to address the missingness in the covariates, which imputed multiple sets (i.e., 10) of missing data based on the observed data and pooled the imputed results together.(17) We presented the baseline characteristics between the SSRI/SNRI and psychotherapy groups using the mean (standard deviation [SD]) for continuous variables and frequency (percentage [%]) for categorical variables. Second, we used multivariable logistic regression to generate the propensity score (PS) of receiving SSRIs/SNRIs vs. psychotherapy (i.e., the conditional probability of receiving SSRIs/SNRIs relative to psychotherapy given a set of covariates including patients’ sex, age, race, region, education, type of insurance, poverty, marital status, physical inactivity, smoking, access to healthcare, severity of depression, comorbidities and comedications mentioned above). Third, we trimmed the analytical cohort using the 5th percentile in the treated group as the lower limit and the 95th percentile in the untreated group as the upper limit.(18) Fourth, we balanced the characteristics between patients receiving SSRIs/SNRIs and those receiving psychotherapy using the stabilized inverse probability treatment weighting (IPTW) approach, which preserves the sample size of the original data and avoids underestimating the variance.(19) Differences in baseline characteristics between the two groups were compared using the absolute standardized mean difference (ASMD), which is considered nonnegligible when ASMD > 0.1. Finally, we performed a multivariable logistic regression using a new diagnosis of dementia (i.e., yes or no) as the dependent variable. In addition to exposure (i.e., receiving SSRI/SNRI vs. psychotherapy), unbalanced covariates (ASMD > 0.1) after IPTW were also included in the regression as independent variables for further adjustment (i.e., doubly robust approach).(20) We reported the crude odds ratio (OR), as well as adjusted OR (aOR) with 95% confidence intervals (CI) after accounting for IPTW to assess the association between SSRI/SNRI use and the risk of dementia using psychotherapy as the comparison group. We also reported the marginal effects of the change in the probability of dementia occurring when using SSRIs/SNRIs compared to psychotherapy after controlling for all other covariates.(21)

To evaluate the heterogeneity in the drug effect among different patient subgroups, we conducted several subgroup analyses by patient sex (i.e., male and female), age group (i.e., < 65 y and ≥ 65 y), race/ethnicity (i.e., white and black), severity of depression (i.e., PHQ-2 score 0–2 and 3–6), concomitant use of non-SSRI/SNRI antidepressants (i.e., yes and no), and underlying cognitive impairment (i.e., yes and no)

## RESULTS

**Figure 2:**
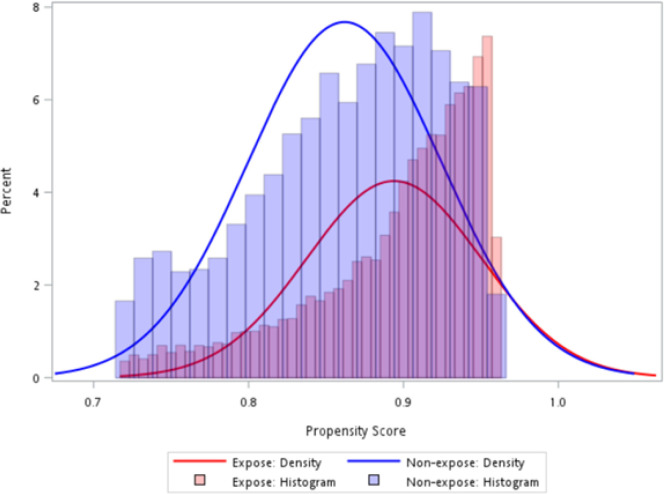
Distribution of propensity scores in the exposed (i.e., SSRI/SNRI) and unexposed (i.e., psychotherapy) groups.

As shown in Fig. 1, a total of 2,710 patients were eligible for the analysis, with 89% receiving SSRIs/SNRIs and 11% receiving psychotherapy. The PSs of the SSRI/SNRI and psychotherapy groups highly overlapped with each other after trimming (Fig. 2), and most covariates were balanced between the SSRI/SNRI users and psychotherapy users (Table 1). The mean age was 60.5 ± 7.8 years for patients using SSRIs/SNRIs and 60.8 ± 8.4 years for patients using psychotherapy. The median PHQ-2 score was 1.4 for both groups. The proportions of patients with underlying cognitive impairment were 20.4% and 21.5% in the SSRI/SNRI and psychotherapy groups, respectively.

In Table 2, the crude incidence of dementia within two years was 16.1% in SSRI/SNRI users and 12.7% in the psychotherapy group. After adjusting for patients’ baseline characteristics, the aOR was 1.39 (95% CI = 1.21–1.59), and the adjusted marginal effect suggested that patients using SSRIs/SNRIs had a 32.6% higher incidence of dementia than those using psychotherapy. Most subgroup analyses reported consistent results with the main analysis, except for patients who were black, had a PHQ-2 score of 3–6, had concomitant antidepressants other than SSRI/SNRI, and had underlying cognitive impairment, for whom the adjusted ORs (95% CI) were 0.75 (0.55–.02), 1.08 (0.82–1.41), 0.75 (0.55–1.02), and 0.84 (0.66–1.05), respectively.

## DISCUSSION

In this retrospective cohort study using nationally representative survey data in the US, we found that older adults with depression receiving SSRIs/SNRIs were associated with a 33% increased risk of dementia within two years compared to those receiving psychotherapy after adjusting for patient characteristics such as age, sex, race, depression severity, underlying cognitive impairment, comorbidities, and concomitant drugs. Most subgroup analyses yielded similar results, except for patients who were black, had a PHQ-2 score of 3–6, had concomitant antidepressant use other than SSRI/SNRI use, and had underlying cognitive impairment, which did not show significant differences in the risk of dementia between SSRI/SNRI use and psychotherapy.

Unlike previous studies largely comparing antidepressant users with nonusers, our study adopted the active comparison group approach to reduce confounding by indication and severity in older adults with depression. Our findings were consistent with some of the prior studies. For example, a meta-analysis including observational studies with at least a 1-year follow-up period showed that SSRI use was associated with an increased risk of dementia compared to no SSRI use, with a pooled risk ratio (RR) of 1.75 (95% CI: 1.03–2.96). However, the heterogeneity in the meta-analysis was extremely high, and one out of five included studies suggested that SSRI use was associated with a lower risk of dementia (RR: 0.58, 95% CI: 0.50–0.68).(22) Another study by Lee et al., restricted to older adults with depression, found that SSRI use was associated with an increased risk of incident dementia, with an adjusted OR of 2.48 (95% CI: 2.27–2.71).(23) Nonetheless, Peakman et al., 2020(24) and Goveas et al., 2012(25) pointed out that even though antidepressant use was found to be associated with the risk of dementia (adjusted hazard ratio [HR]: 1.32 [95% CI: 1.01–1.74] and 1.69 [95% CI: 1.21–2.35], respectively), an association was not found for SSRIs (adjusted HR: 1.07 [95% CI: 0.91–1.25] and 1.50 [95% CI: 0.89–2.53], respectively). Instead, this association may be attributed to TCAs, which were found to be associated with incident dementia (adjusted HR: 1.75 [95% CI: 1.05–2.91] reported by Goveas et al., 2012). Other reasons contributing to the conflicting findings from the existing studies include prior claim-based studies unable to include the severity of depression and cognitive impairment status in the analysis. Our survey data analysis addressed these issues by including patients’ PHQ-2 score, couse of other antidepressants, and underlying cognitive impairment.

In the subgroup analyses, we found that the association between SSRI/SNRI use and dementia risk did not exist in patients with a higher PHQ-2 score, concomitantly receiving non-SSRI/SNRI antidepressants, or with underlying cognitive impairment. Patients with a higher PHQ-2 score or concurrently used non-SSRI/SNRI antidepressants might reflect those with more severe depression or uncontrolled depression, which may confound the drug effect. (26) In addition, the use of other antidepressants with a larger anticholinergic burden, such as TCAs (27), and the progression of depression(28) may play a critical role in the risk of dementia, which may mask the effect of SSRIs/SNRIs.

The association also did not exist in patients with underlying cognitive impairment, which is probably because clinicians are more concerned about the risk of dementia if patients have prior cognitive impairment, and thus psychotherapy is preferred to SSRI/SNRI use.(22) However, this group of patients is at high risk of dementia, which may lead to mitigation of the risk in the SSRI/SNRI group. The association also did not exist in Black people, probably because Black people are less likely to receive SSRI/SNRI than White people even though they have similar severity of depression,(29) which may dilute the drug effect as well. Finally, the reason for no association among these subgroups could also be due to the small sample sizes after stratification.

There are some limitations in our study. First, we used MEPS data, which only follow a patient for at most 2 years, which may not be long enough for dementia to occur and underestimate the risk of dementia.(30) However, in a population-based study with a mean follow-up of 8 years, the incidence of dementia was 13% among older adults with depression,(31) similar to our findings. Second, we were unable to identify incident new users of SSRIs/SNRIs and psychotherapy due to the lack of a washout period. Therefore, we could not address the depletion of susceptibles,(32) meaning that patients who were using SSRI/SNRI may be the ones who were less likely to incur dementia. Third, although we conducted several subgroup analyses to address potential heterogeneity in the drug effect across patient subgroups, we were unable to include potential confounders such as duration of depression(29).

## CONCLUSION

Our findings provide valuable insight into the complex association among depression, antidepressants, and risk of dementia, providing some guidance for clinicians while prescribing antidepressants for patients with depression. Future longitudinal studies are warranted to allow the identification of new users of antidepressants and the evaluation of long-term dementia risk.

## Figures and Tables

**Table 1:** Baseline characteristics of the study population: 2010–2019 Medical Expenditure Panel Survey Data

	Unweighted population (n = 2,710)			Weighted population (n = 1,858)		
	SSRIs/SNRIs (n = 2,402)	Psychotherapy (n = 308)	ASMD	SSRIs/SNRIs (n = 1,652)	Psychotherapy (n = 206)	ASMD
Age, mean (SD)	63 (9.2)	59.2 (8.0)	0.43*	60.5 (7.8)	60.8 (8.4)	−0.03
PHQ-2, median (IQR)	1.4 (1.7)	1.8 (1.9)	0.21*	1.4 (1.8)	1.4 (1.8)	0.04
Cognitive impairment, n (%)	451 (18.8)	90 (29.2)	0.25*	337 (20.4)	44 (21.5)	−0.03
Female, n (%)	1688 (70.3)	187 (60.7)	0.20*	1133 (68.6)	143 (69.3)	−0.02
Race, n (%)						
White	1783 (87.0)	211 (78.7)	0.22*	1404 (84.9)	170 (82.3)	0.07
Black	161 (7.9)	42 (15.7)	0.24*	153 (9.3)	26 (12.7)	−0.11*
Others	106 (5.2)	15 (5.6)	0.02	96 (5.8)	10 (5.1)	0.03
Hispanic, n (%)	271 (11.3)	34 (11.0)	0.01	196 (11.9)	24 (11.8)	0
Region, n (%)						
Northeast	356 (14.8)	85 (27.6)	0.32*	286 (17.3)	33 (16.2)	0.03
Midwest	593 (24.7)	79 (25.6)	0.02	446 (27)	55 (26.5)	0.01
South	922 (38.4)	74 (24.0)	0.31*	512 (31)	71 (34.5)	−0.07
West	531 (22.1)	70 (22.7)	0.01	409 (24.7)	47 (22.8)	0.05
Education, n (%)						
No degree	284 (15.4)	29 (11.9)	0.10	223 (13.5)	32 (15.5)	−0.06
General education development	87 (4.7)	11 (4.5)	0.01	77 (4.7)	11 (5.4)	−0.03
High school	832 (45.0)	82 (33.6)	0.24*	657 (39.7)	74 (36.1)	0.08
Higher education	473 (25.6)	83 (34.0)	0.18*	509 (30.8)	67 (32.5)	−0.04
Others	171 (9.3)	39 (16.0)	0.20*	186 (11.3)	22 (10.6)	0.02
Poverty, n (%)						
Poor/negative	335 (13.9)	67 (21.8)	0.20*	252 (15.2)	33 (16.2)	−0.03
Near poor	133 (5.5)	17 (5.5)	0.00	83 (5)	12 (6)	−0.04
Low income	358 (14.9)	47 (15.3)	0.01	247 (15)	34 (16.6)	−0.05
Middle income	716 (29.8)	65 (21.1)	0.20*	421 (25.5)	54 (26.1)	−0.01
High income	860 (35.8)	112 (36.4)	0.01	650 (39.3)	72 (35.1)	0.09
Marital status, n (%)						
Married	1323 (55.1)	131 (42.5)	0.25*	882 (53.4)	107 (51.7)	0.03
Separated, Widowed or Divorced	175 (7.3)	48 (15.6)	0.26*	131 (7.9)	17 (8.1)	−0.01
Never married	904 (37.6)	129 (41.9)	0.09	639 (38.7)	83 (40.2)	−0.03
Physical inactivity, n (%)	891 (37.1)	119 (38.6)	0.03	600 (36.3)	81 (39.4)	−0.06
Smoking, n (%)	356 (18.5)	52 (21.9)	0.09	325 (19.7)	30 (14.8)	0.13*
Limited access to health care, n (%)	224 (12.9)	36 (16.9)	0.11*	242 (14.6)	26 (12.4)	0.06
Comorbidities, n (%)						
Cancer	14 (0.6)	2 (0.6)	0.01	9 (0.5)	2 (0.9)	−0.04
Type 2 diabetes	41 (1.7)	5 (1.6)	0.01	30 (1.8)	5 (2.4)	−0.04
Hyperlipidemia	67 (2.8)	13 (4.2)	0.08	57 (3.5)	12 (5.9)	−0.11*
Hypertension	61 (2.5)	7 (2.3)	0.02	42 (2.6)	7 (3.5)	−0.05
Ischemic stroke	1 (0)	0 (0)	0.03	0 (0)	0 (0)	0
Chronic heart disease	18 (0.7)	1 (0.3)	0.06	9 (0.5)	1 (0.7)	−0.02
Osteoarthritis	29 (1.2)	3 (1)	0.02	19 (1.2)	1 (0.6)	0.07
Parkinson’s disease	1 (0)	0 (0)	0.03	0 (0)	0 (0)	0
Anxiety	373 (15.5)	27 (8.8)	0.21*	177 (10.7)	15 (7.3)	0.12*
Sleep disorder	23 (1.0)	6 (1.9)	0.08	19 (1.1)	2 (0.8)	0.03
Schizophrenia	0 (0)	3 (1.0)	0.14*	0 (0)	0 (0)	0
Bipolar disorder	14 (0.6)	14 (4.5)	0.25*	6 (0.3)	0 (0.1)	0.06
Comedications, n (%)						
Analgesics	201 (8.4)	27 (8.8)	0.01	146 (8.9)	20 (9.5)	−0.02
Benzodiazepines	56 (2.3)	14 (4.5)	0.12*	43 (2.6)	6 (3)	−0.02
Anxiolytics, sedatives, and hypnotics	62 (2.6)	6 (1.9)	0.04	40 (2.4)	7 (3.3)	−0.05
Antidepressants other than SSRI/SNRI	46 (1.9)	12 (3.9)	0.12*	33 (2)	3 (1.4)	0.04
Antipsychotics	13 (0.5)	17 (5.5)	0.29*	2 (0.1)	1 (0.4)	−0.06
Antiparkinsonian agents	15 (0.6)	4 (1.3)	0.07	13 (0.8)	0 (0)	0.13*

Abbreviation: SSRI: selective serotonin reuptake inhibitors, SNRI: serotonin and norepinephrine reuptake inhibitors, SD: standard deviation, ASMD: absolute standardized mean difference

* ASMD > 0.1 means there’s a significant difference in the variable between the two groups

**Table 2:** Odds ratios of dementia among older adults with depression using selective serotonin reuptake inhibitors (SSRIs)/serotonin and norepinephrine reuptake inhibitors (SNRIs) compared to those receiving psychotherapy

	Crude incidence (%)		OR (95% CI)		Adjusted marginal effect (%)
	SSRI/SNRI	Psychotherapy	Crude	Adjusted	
Main analysis: SSRI/SNRI vs. psychotherapy (n = 2,710)	16.1	12.7	1.46 (0.97–2.20)	1.39 (1.21–1.59)*	32.6*
Subgroup analysis stratified by					
Age					
< 65 years (n = 1,697)	15.3	12.3	1.18 (0.75–1.87)	1.38 (1.17–1.64)*	32.4*
≥ 65 years (n = 1,013)	17.3	13.9	2.38 (1.05–5.41)	1.52 (1.21–1.91)*	41.8*
Sex					
Male (n = 835)	16.5	11.6	1.42 (0.81–2.50)	1.37 (1.08–1.74)*	31.4*
Female (n = 1,875)	15.9	13.4	1.52 (0.91–2.54)	1.40 (1.19–1.65)*	33.4*
Race					
White (n = 1,994)	14.0	9.0	1.81 (1.00–3.27)	1.60 (1.36–1.87)*	46.8*
Black (n = 203)	21.1	16.7	1.04 (0.62–1.74)	0.75 (0.55–1.02)	−28.6
PHQ-2 score					
0–2 (n = 1,918)	15.6	12.1	1.39 (0.83–2.33)	1.49 (1.28–1.75)*	40.1*
3–6 (n = 506)	19.3	15.4	1.75 (0.96–3.20)	1.08 (0.82–1.41)	7.2
Concomitant antidepressants other than SSRI/SNRI					
Yes (n = 58)	28.3	16.7	7.67 (6.93–8.49)	0.75 (0.34–1.66)	−28.3
No (n = 2,652)	15.8	12.5	1.41 (0.94–2.13)	1.40 (1.22–1.61)*	33.7*
Underlying cognitive impairment					
Yes (n = 541)	22.8	17.8	1.49 (0.99–2.24)	0.84 (0.66–1.05)	−17.9
No (n = 2,169)	14.5	10.6	1.51 (0.89–2.57)	0.78 (1.50–2.12)*	57.7*

Abbreviations: SSRI, selective serotonin reuptake inhibitors; SNRI, serotonin and norepinephrine reuptake inhibitors; RRD, relative risk difference; OR, odds ratio; CI, confidence interval

* An asterisk means that the result is significant

## Data Availability

All data analyzed in this article are publicly available at https://www.meps.ahrq.gov/mepsweb/data_stats/download_data_files.jsp

## IMPACT STATEMENT

We certify that this work is novel. This work is the first to compare first-line antidepressants (i.e., SSRI/SNRI) to an active comparator (i.e., psychotherapy) to reduce confounding by indication instead of comparing antidepressant use to no use in prior studies.

## Key Points

- Our findings suggested that SSRI/SNRI use was associated with an increased dementia risk compared to psychotherapy among older adults with depression.
- Subgroup analyses yielded similar findings as the main analyses, except no significant association for patients who were black, had a higher PHQ-2 score, had concomitant non-SSRI/SNRI antidepressants, and had underlying cognitive impairment.

## Why does this matter?

Clinicians should be aware of SSRI/SNRI-associated dementia risk when considering the first-line treatment for older adults with depression. Patient characteristics may also play a critical role in such an association.
